# Indents

**Published:** 1911-09-15

**Authors:** 


### INDENTS.

☞ **Brief Articles of Technical or Scientific Value will receive notice and comment. Contributions invited.**

*To Wet Wheel While Grinding Teeth.* To keep stone wet while ginding teeth, affix a small piece of sponge by means of a rubber band to the middle finger. This is easily held in contact with the wheel and keeps wheel wet as well as clean.—R. H. Daniels, Peoria.

*Stimulant, Diffusible.* As a stimulating and soothing application in cases of pericementitis, and in pyorrhea treatment, the following painted on the gums with a camel-hair brush every two or three hours will be found very beneficial, and may be used by the patient:

| | | |
|---|---|---|
| ℞ | Tincture iodin | ℥ss |
| | Tincture aconite | |
| | Chloroform | aa fl. ℥j |
| | Glycerin | ℥j |
| | Peppermint | gtt. v |
| | M. | |

—Dr. H. C. Register, *Dental Cosmos*, September, 1910.

*To Make Pink Gum Rubber Look Natural.* Moisten a piece of cotton with sulfuric acid and rub it over the pink rubber until it turns black. Then wash thorougly to remove all traces of the acid, and it will be found that the artificial gum has become a perfect imitation in color of the natural gum. Another method is to allow the denture to lie in a covered glass jar filled with methylated spirit, and expose it to the sunlight. It will be found that the pink rubber becomes paler and paler according to the length of time of exposure, and also the strength of the sunlight. The denture is removed when the desired shade of color is obtained.—*Chemist and Druggist.*

*Use of Novocain in Solutions of Sodium Bicarbonate and Salt for Local Anesthesia.* Laewen, by experiments with novocain chlorid and novocain bicarbonate, has shown that the terminal filaments of the sensitive nerves are acted upon by solutions of the bicardonate of novocain of one-half to one-third the strength required by solutions of the chlorid. He also finds that the anesthesia produced by the bicarbonate lasts considerably longer than that produced by the chlorid.—*Muenchener Med. Wochenschrift* per *New York Med. Journal.*

*How to obtain a smooth, dense surface on Casts.* To insure smooth, dense surfaces on plaster casts, also on casts made of investment materials, we recommend that impressions of all-plaster be used; or, if modeling compound or wax be used, that a thin layer or coating of plaster be applied to the surface of the impression.

To prepare the impression for pouring a model of model-plaster (New York plaster), for vulcanite work, first give the impression a thin coat of shellac varnish. When this has become dry, which it will in about thirty minutes, give the impression a thin coat of sandarac varnish. This also should be given time to harden, about twenty minutes after which we place the impression in water and allow it to become saturated before pouring the model. As an experiment, take any piece of plaster that has been lying around the laboratory for a few hours, and put it in water; bubbles will rise on the surface very freely, even through a varnished surface. Now, if freshly-mixed plaster or investment material were placed in a dry impression, the same thing would occur; bubbles would come from the surface of the dry plaster and a model full of holes, forced in by these bubbles of gas, would be the result. The reason for using the varnishes, as stated, is that the shellac acts as a filler on the fresh plaster surface, just enough being used to fill the pores and stain the plaster to a slight depth, leaving the surface, so far as the fine lines of the impression are concerned, just as it was before varnishing.

If the impression were to be poured at this stage, with just the shellac as a separating medium, there would be difficulty in separating, as the plaster, in setting, creates enough heat to soften the shellac and thoroughly glue the two parts together.

Some are in the habit of using oil or soap to prevent this stickingtogether of the impression and model, but this is not good practice, as the oil or soap will unite with the fresh plaster, and a very poor surface on the model is sure to be the result. So we use sandarac varnish, thin sandarac varnish, as a second coat on the impression. All that is needed is just enough to give a smooth, glossy surface. We prefer a colored varnish, as it is easier to ascertain just how much is being used.

When the varnish has hardened, place the impression in water and leave it there until saturated, so that no bubbles of gas will rise up and fill the surface of model with the holes that we all have seen, and so that the dry plaster will not take up some of the necessary water from the fresh plaster, and give us a porous surface of poor quality.

Sandarac varnish is practically inert so far as the pouring of impressions is concerned and can be separated by carving away the impression over the ridge until the stain made by the shellac appears; then place the point of a knife under the edge and gently break away from undercuts and around teeth.

After separating the impression, the first thing to do is to give the surface of the model a good coat of castgloss, or silex, before anything else gets onto the surface. Hot wax or grease of any kind, if run over the surface before applying silex preparation, will prevent the plaster from taking up the silex, and a rough, porous surface on the palate of the plate will result.

After applying castgloss, wipe the model dry with a piece of cotton, rubbing hard enough to polish the surface somewhat. Hot wax or any other material may now be run over the surface of the model, and the plate will still come out with a smooth, clean surface.—Dr. F. L. Olds in *Dental Summary*.

*Soldering.* It must be borne in mind that to effect a union between two metals these must be free from any substance which heat will not remove. If the surfaces of metals are oxidized, the molecules will not unite. As the gold plate we use is never pure, and as the solder used is less pure than the plate, both would oxidize under heat and fail to unite. To prevent this a flux is used, which is generally borax, either alone or in combination. such as—

℞—Boric acid, ℥ss

Muriate ammonia, gr. xj

Carb. potassium, gr. v

Borax pulv., ℥ss

Aq., q.s. M.

When the surfaces to be united are coated with a flux and heated, the borax fuses and spreads over the surface in the form of a "glass," thus keeping the air from coming in contact with the parts, and preventing oxidation. The borax probably plays a minor part in dissolving any oxidation that previously may have existed on the surfaces. Too much borax will cause the solder to flow beyond its intended limit, while too little will not entirely prevent oxidation. It is not, however, the process of uniting metalic surfaces which brings trouble to the dentist, but the act of soldering metals in the presence of porcelain. Metals may be heated to any point short of fusion, slowly or quickly, without materially affecting the process of soldering, but where porcelain is present it must be both heated slowly and cooled slowly, in order to avoid checking. When metals and porcelain are in contact, the difficulty of heating both uniformly is greatly increased, because one is a good conductor of heat, while the other is a poor one. If the temperature be raised much in advance of the porcelain, the latter will be fractured by too rapid expansion. To avoid this, it becomes necessary to incase them in a substance which will both protect them from direct flame and insure gradual heating. The enveloping material must be such as not to be affected by heat, and of sufficient quantity to allow the heat to reach the porcelain equally and slowly. Sea sand, pumice stone, and marble dust, or pulverized asbestos, usually form the bases of an investment, while plaster of paris is used to hold the material together, and form a mass which will retain the teeth and plate in proper relation to one another during soldering, as plaster is liable to crack and change its form under heat. Only enough should be used to hold the other substances together. Excess of plaster is the cause of cracking of an investment, which is also frequently attended by fractures of the porcelain. Sea sand, 4 parts; plaster, 1 part; large investment—pumice, plaster, equal parts. After the hard wax which holds the parts in position has been removed, the pins and all exposed metals should receive a slight coating of borax. The investment should then be placed on a slow fire and heated well. The solder is cut into pieces about one-eighth inch square and dipped in borax. In bridge work a small quantity of solder should be used first, as it is more likely to find its way into obscure places. After the first portion has been melted and flowed into deeper parts, more may be added, and the soldering completed. In this way more perfect results will be obtained. In all cases the investments should be placed so that the teeth are heated first; the porcelain then expands and also allows the platinum pins to do likewise. The flame should not be placed upon after the backing until the solder shows signs of melting, while a fine flame should be kept on the solder till it flows. Pins should not be riveted but bent for holding backing in place. The piece should be covered immediately after the soldering is finished, for uniform cooling. R. Sutherland, *Commonwealth Dental Review.*

*Dental Laboratory Fire Risks.* Recently, the contents of a dental office were destroyed by a fire originating from the vulcanizer gas-burner. The room was in an office building, and was divided by partitions, as many such rooms are, into operating, waiting, and work-room. The dentist was at work until late in the evening and forgot, when leaving, that his vulcanizer was in operation. Later, it is supposed that the rubber gas tube either leaked or slipped from the burner, and the escaping gas igniting, set fire to the work bench, and this in turn to the partitions and furniture. The building being fireproof, confined the flames to the one room. While the contents of the room were ruined, sufficient was left intact to show how the fire originated. All gas-using appliances that are frequently in use for a long time should have metal connections extending sufficiently far from the gas flame as to make improbable a fire from a defect in the rubber hose. The work-bench on which they are used should be covered with an asbestos board, and the space partitioned off for a work-room will be safer from fire if lined with asbestos cloth. It is inexpensive, and not unsightly. Rubber hose of the present day is not at all reliable, it may become hard and break, it may soften by heat, or become porous. A timing arrangement for the vulcanizer to shut off the gas when vulcanizing is complete makes matters safer for the work, and the work-room. A dentist who had his electric porcelain furnace neatly installed in a cabinet which when closed kept it and its belongings from the dust and dirt of the work-room, made a narrow escape from fire through closing the cabinet without opening the switch cutting off the current. These are matters calling for an ounce of prevention.—Trueman in *Brief*.

*Protection of the Dental Pulp Under Silicate Cement Fillings.* The underlayings of silicate cement fillings with Fletcher's or oxyphosphate cement in the author's opinion is contra-indicated, since these materials occupy too much space. Instead, a small piece of gold foil No. 30 is recommended, as this material is impervious to chemical influences and occupies an infinitesimally small space, so that it can be burnished even into the under cuts without detriment. Moreover, this method of procedure requires very little time and recommends itself aseptic by its nature. The working method is as follows: A piece of gold foil of about the size of the cavity is firmly pressed against the cavity walls and into the undercuts by means of small pieces of punk, any excess either to be cut off with a sharp instrument or to be bent inwardly and again firmly pressed against the cavity walls. Silicate cement is then pressed against the gold foil and the margins under strong pressure, and burnished with small ball burnishers If, in exceptional cases the foil should not hold in the cavity, a trace of mastic or gutta-percha is first inserted into the cavity and the gold foil is burnished in with a warmed instrument. The transparency of the silicate cement filling is considerably enhanced by the gold foil matrix, a tin foil matrix also serving a good purpose, but not affording the same degree of transparency. Secondary caries is retarded and thermal irritation, so often experienced in gold fillings, is prevented.

A thorough critical review of the periodical literature on this subject and the histories of practical cases by Dr. Felier strongly argue in favor of the author's method of pulp protection.—Dr. Erich Feiler, *Dental Cosmos.*

*Treatment of Sensitive Cervical Cavities.* I prepare sensitive cervical cavities with the least pain of any method of which I am aware. Nitrate of silver is good and I use it very much indeed. Anyone who is engaged considerably with the treatment of pyorrhea must very often use nitrate of silver or some other method to relieve that extraordinary sensitiveness following an operation.

When there are cavities at the cervical third, if I desire to fill immediately or as soon as possible, my plan is to have the patient cleanse, with violent rinsing, the mouth, commencing with warm water with a good lot of salt in it, then gradually increasing the heat of the water until it gets up to about 135 or 140 degrees, and after the patient has finished this thorough rinsing, you supplement it with an application of hot water from the syringe with force, flushing the margins again and again until all extraneous deposits are removed. This very hot water is of itself one of the best obtundents, preceding all others. Then I sometimes use a strong, saturated solution of cocaine upon these surfaces, pressing in with a body of unvulcanized rubber, just as we do when pressing into a pulp; to prepare these surfaces for filling, when they need filling, I find nothing so good as to use oxychloride of zinc, not excavating but very slightly going over them with a polishing powder and wood stick, drying the surfaces, putting in a pad or roll of cotton one side or the other to keep away moisture, covering all those sensitive surfaces with the chloride of zinc—not the oxyphosphate. Slap it in between the teeth and over those surfaces, keeping it free from moisture a few minutes until dry and let it remain there three or four days. I know of nothing which is so simple and cheap which affords such perfect immunity to any sensitiveness and which permits you to go on and fill the tooth by any material you choose to apply. I use that on sensitive surfaces after an operation for pyorrhea, when the labial surfaces of anterior teeth and the proximal surfaces are extremely sensitive. I mix up a batch of zinc, keep the saliva away and thoroughly cover the surfaces, and in three or four days you can pick it out readily, and you will find it has relieved that sensitiveness completely. There is no pain inflicted after the preparatory treatment is finished.

The oxychloride of zinc is a very quick setting material and will harden in a few minutes. You can press it over the surface with the ball of your thumb or finger and do no other finishing at all.—Dr. J. D. Patterson in *Western Journal.*

